# Oral hygiene improvement: a pragmatic approach based upon risk and motivation levels

**DOI:** 10.1186/1472-6831-8-31

**Published:** 2008-11-11

**Authors:** Harold D Sgan-Cohen

**Affiliations:** 1 Department of Community Dentistry, Hebrew University-Hadassah School of Dental Medicine, Jerusalem, Israel

## Abstract

Good oral hygiene has always been the cornerstone of public and private dental health promotion. However, this has often been based upon incorrect assumptions. The public is not always willing and does not always need to change its oral health behavior to the same extent as that expected by the dental profession. The present commentary emphasizes the need to modify oral hygiene instruction according to specific risk and motivation levels. Dentistry needs to be flexible in accepting new evidence-based modalities of oral health promotion. Dentists, dental hygienists and the entire health care team need to accept that the traditional methods of oral health education are not always effective.

## Article

The single most continuous theme of preventive and public health dentistry has been and remains the cleaning of teeth [1]. In the present commentary "clean teeth" will be equated to good oral hygiene and/or low dental plaque levels. This simple objective has been difficult and at times not even possible to attain. The fundamental importance of oral hygiene, moreover, has often been controversial. Diocletian Lewis (1823–1886), an illegal medical practitioner, coined the adage "A clean tooth never decays". Over the years, nevertheless, this has been heatedly contested. Many authors have advocated a narrow association between dental plaque levels and gingival disease. However in 1998, the European Workshop on Mechanical Plaque Removal clearly stated that "Forty years of experimental research, clinical trials and demonstration projects in different geographical and social settings have confirmed that effective removal of dental plaque is essential to dental and periodontal health throughout life" [2]. This statement, based upon an overwhelming abundance of evidence, is today commonly accepted.

If we accept that cleaning teeth is an imperative public oral health requirement, we need to explore the ways and means of achieving this goal. How can we assist, facilitate, motivate and even educate people to clean their teeth? I strongly believe that it is considerably easier to treat caries (but less important).

Traditionally, preventive dentistry has correctly assumed that most oral diseases are preventable and effective methods (including oral hygiene) have been revealed. The profession has correctly acknowledged that applied prevention is not always easy and demands optimal cooperation and motivation of the public. Preventive dentistry has incorrectly assumed that: knowledge and information promote health; patients more often than not want to change their behaviors; patients more often than not need to change their behavior; patients believe that oral health is of the utmost importance; patients are motivated to do what we tell them; health promotion demands the modification of all health behaviors. Traditional health education has been exceptionally unsuccessful in modifying health behavior and health, as demonstrated by Kay and Locker [3] and Watt [4].

The foundation for any health intervention, including oral hygiene instruction, should be firmly based on scientific evidence and not on tradition alone. Two imperative components, which should be recognized, are the risk and motivation of the patients and their communities.

Over the last few years the concept of Caries Management Based on Risk Assessment (CAMBRA) has been widely adopted and applied to dental practice. This has been clearly presented and described in an applicative pragmatic manner. Featherstone, in 2004, has stated that "A structured caries risk assessment should be carried out based upon the concept of the caries balance. Following the risk assessment a treatment plan is devised which leads to the control of dental caries for the patient" [5]. The "caries management system" has been reiterated and explained by other authors [6]. Low risk patients are classified, among other criteria, as those with low plaque levels. Medium risk patients are those with medium plaque levels and high risk patients are those with abundant plaque levels. Orthodontic treatment should not be forgotten in this equation. Low risk patients would include those with minor orthodontic treatment: short term removable appliances; medium risk would include patients undergoing longer term orthodontic treatment, usually with active fixed appliances; high risk patients would include complex cases (e.g. skeletal malocclusion, anatomical oral malformations, etc.) and long term treatment with active fixed appliances, often including surgical or prosthetic involvement. Orthodontic appliances and brackets should be fully recognized as plaque retaining vehicles.

Preventive treatment/management/care should be based on and not independent of these risk levels. Care for low risk groups would essentially concentrate on maintenance and reinforcement of existing oral hygiene practices. For medium risk groups more recall and utilization of fluorides should be recommended and for high risk groups recall with dentists and hygienists and maximal utilization of fluorides and anti-microbial agents, could be recommended. Among medium and high risk groups full consideration should be placed upon postponing or even canceling clinical treatment until further notice (unless there is a clear and urgent medical indication).

It needs to be impressed that we should not always re-educate or supply an abundance of often unnecessary dental health knowledge. Patients with clean and healthy teeth and gingival tissue are not required by any rationale to be optimally familiar with dental anatomy, histology, microbiology etc. Almost 30 years ago Frazier and Horowitz correctly affirmed that "Those responsible for recommending and implementing community-based oral health programs must be conscientious, ethical, and flexible, they must be willing to accept new scientific evidence" [7]. Nevertheless and sadly, significant numbers of dentists and hygienists cannot resist providing tremendous amounts of "dental knowledge", even before adequate individual or community diagnoses and risk levels have been defined. We have adopted and recommended a pragmatic approach for schoolchildren, with the focused objective of modifying brushing skills, without excessive elaboration of dental knowledge education [8].

Finally, the motivation of individuals and communities should be optimally explored and understood. The most difficult patients are often, but not always, those at the highest risk with the lowest motivation. On the other hand, there are many individuals and communities at low risk with high motivation levels. This situation emphasizes the need to negate the common attitude of a universal "treatment plan". The recently developed "Motivational Interviewing" (MI) is designed as a brief, non-confrontational, technique of facilitating making changes in behavior. MI accepts the patients' existing motivation and aims to amicably help choose an optimal behavior based upon the existing motivation. MI does not tell people what to do and fully recognizes that change is not entirely easy. This method has been effectively utilized in physical exercise, smoking, alcohol abuse, oral health and other behavior modifications [9,10].

Dental health practitioners should recognize the unique fabric of each individual within his or her community. All people and all communities are not the same. We are all social beings. A person within a community that does not floss will not floss. A person within a community that regularly visits dental hygienists will follow suite. A person within a community that consumes massive amounts of candy will do the same – even if the dentist or hygienist "tells them" otherwise. We are not all the same. Some groups of people need our assistance, some do not. Some people want our assistance and some do not.

Among those members of the health care team, who are committed to oral health promotion, there is no need for complacency but also not for despondency. After all, improved tooth cleaning can and should be a realistic and not an exaggerated expectation.

## Competing interests

The author declares that they have no competing interests.

## Pre-publication history

The pre-publication history for this paper can be accessed here:


